# Integrating computer vision and molecular neurobiology to bridge the gap between behavior and the brain

**DOI:** 10.1016/j.cois.2024.101259

**Published:** 2024-09-05

**Authors:** Ian M Traniello, Sarah D Kocher

**Affiliations:** 1 Lewis-Sigler Institute for Integrative Genomics, Princeton University, Princeton, NJ, USA; 2 Department of Ecology and Evolutionary Biology, Princeton University, Princeton, NJ, USA

## Abstract

The past decade of social insect research has seen rapid development in automated behavioral tracking and molecular profiling of the nervous system, two distinct but complementary lines of inquiry into phenotypic variation across individuals, colonies, populations, and species. These experimental strategies have developed largely in parallel, as automated tracking generates a continuous stream of behavioral data, while, in contrast, ‘omics-based profiling provides a single ‘snapshot’ of the brain. Better integration of these approaches applied to studying variation in social behavior will reveal the underlying genetic and neurobiological mechanisms that shape the evolution and diversification of social life. In this review, we discuss relevant advances in both fields and propose new strategies to better elucidate the molecular and behavioral innovations that generate social life.

## Introduction

Complex social organization, exemplified by the eusocial societies of ants, bees, wasps, and termites, has greatly facilitated the success and diversification of these insect groups [1]. Though we understand many of the ultimate factors that have enabled social evolution, we do not yet have a clear understanding of its mechanistic basis, particularly how the nervous system supports social life in a group or colony. Social insects have tiny yet fundamentally heterogenous and complex brains [2], the genetic architecture of which can now be profiled at single-cell and spatial resolution [3]. Similarly, analogous advances in automated tracking have made it possible to generate a lifelong record of every physical thing each insect in a colony does [4], overcoming observational challenges that plague standardization even in more traditional ‘model’ systems [5]. These advances allow us to bring higher resolution to classic questions in neuroethology as well as to ask new questions regarding how behavior should be defined and quantified and how high-dimensional behavioral data sets can be interpreted in light of molecular methods that provide a ‘snapshot’ of brain activity.

In this article, we review relevant technological advances in behavioral and molecular profiling of social insects and their brains, and we discuss strategies to better integrate these distinct but complementary lines of experimental inquiry.

## Rationale for automated tracking of social insects

Within the colony, phenotypic variation among nest-mates arises from a shared genome that generates diverse morphological and behavioral traits that compose the group. Over a century of observational ethology has elegantly characterized behavioral mechanisms of co-operation, interactivity, social dominance, and other higher-order features that define social insect societies [6,7]. Organizing features of eusociality such as division of labor and caste-specific behavioral repertoires have been described without the need for automation, yet even the most robust behavioral classifications can benefit from increased resolution of behavioral profiling.

**Figure 1:**
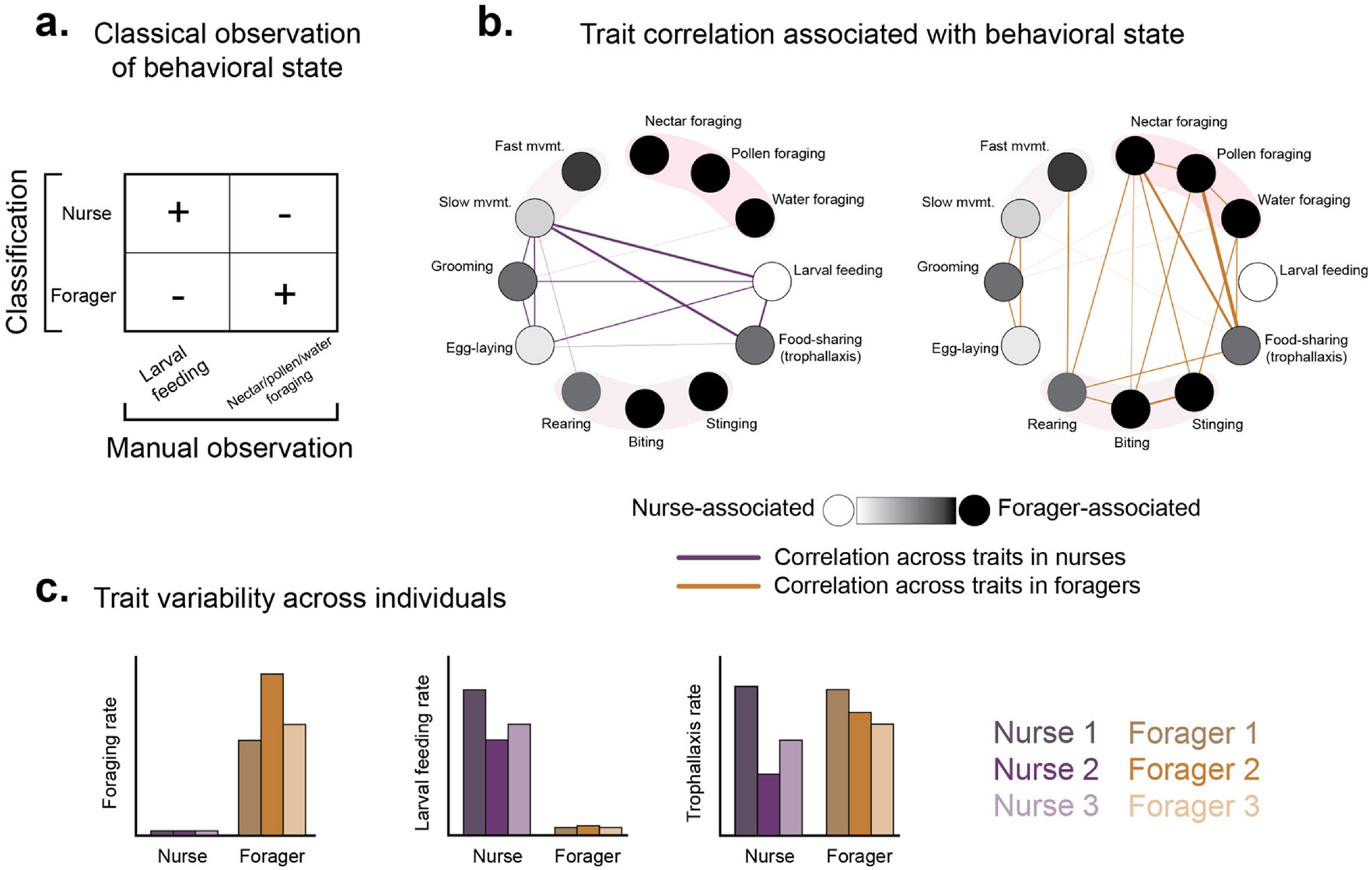
Manual versus automated classification of behavioral state. **(a)** Honey bee (*Apis mellifera*) nurses and foragers can be classified generally by the presence or absence of manually observed behaviors like larval feeding or out-of-hive foraging, yet **(b)** each state is made up of a suite of correlated traits that are difficult or impossible to manually observe, and **(c)** substantial variability in state-defining traits exists across individuals. In this example, figures are based on theoretical data informed by recent studies that have leveraged automated tracking to explore division of labor in honey bee colonies [19,53,70]. Shaded bands that group circles together in **(b)** represent associated or related traits.

For example, young honey bee workers robustly perform in-hive tasks such as feeding larvae (‘nursing’) before transitioning to out-of-hive tasks such as colony defense and foraging later in life (Figure 1a). Nurse and forager bees are distinguishable by age, physiology, and behavioral repertoire [8], and molecular profiling of brain gene expression has identified strong neuromolecular correlates of each behavioral state [9]. Yet, each state is characterized by a constellation of behavioral and physiological traits that can strongly vary across individuals [10], colonies [11,12], populations [13], and timescales [14], even as the behavioral phenotype remains classifiable in generalized behavioral and molecular terms (Figure 1b,c). In this regard, ‘behavior’ describes a suite of correlated traits, most of which are obscured from manual observation but detectable via automated tracking. Therefore, in addition to being a boon for ethological research, automated tracking interrogates fundamental assumptions about how behavior is classified.

## Automated tracking to discern the structure of social insect behavior

The past decade has seen tremendous advancement in the capacity to study social insect behavior under naturalistic conditions in the laboratory and field. Where limitations based on observer attendance and bias once dominated, the scale and resolution at which individual and collective behavior can now be monitored have greatly benefited from improvements in machine vision and engineering, fields that have been synthesized to promote the new discipline of computational ethology [4,15,16]. These novel monitoring approaches allow users to quantify spontaneous and social behaviors among freely moving animals that were previously impossible to identify via human observation [17,18], with far-reaching ramifications for our understanding of division of labor [19,20], pollinator health [21,22], and behavioral evolution [23].

We argue that most ethological investigations concerning physical behaviors can be addressed by any tracking system that meets two basic requirements. The first requirement is preservation of individual identity independent of group or colony size. This requirement is satisfied via the use of a fiducial marker, a unique ‘tag’ typically consisting of either a barcode or a paint mark, which represents an individual’s identity for the duration of an experiment. In addition to identity, a fiducial marker can be tracked as an individual’s centroid, allowing for position, linear and angular speed, and other kinematic metrics to be measured [22].

The second requirement is more complex: computer vision-based identification of each animal’s physical attributes (i.e. antennae, body segments, legs, etc.) in space. This process, deployed by tools such as SLEAP [24] and DeepLabCut [25], is generally referred to as pose estimation, with *pose* referring to an animal’s position and orientation in space, and *estimation* meaning that deep learning is used to predict the location and position of each animal’s body parts; we refer to the representation of body parts by anatomy-defining nodes and edges as an animal’s *skeleton*. In pose estimation, the skeleton is typically defined by the user and can scale in terms of anatomical complexity to address specific questions. This requirement gives the user flexibility to perform tailored analyses of how an individual moves and interacts with their environment.

Building on these concepts, most tracking software can be classified as marker-based (i.e. uses a fiducial marker) or markerless. Markerless approaches are less invasive and avoid the need to immobilize an insect for barcode placement, thus reducing time investment and handling stress for the investigator and animal, respectively. Tools such as idTracker.ai [26] and TRex [27], which can simultaneously track dozens of unmarked individuals, function by first identifying ‘blobs’, or areas of a video frame that contain either one or more animals, before applying a convolutional neural network (NN) to determine the specific number of individuals in a given blob. This process is straightforward and efficient for tracking location and movement, but these tools struggle to ascertain unique identities if individuals are visually indistinguishable or prone to occluding one another, as is frequently the case for social insects.

In contrast, marker-based tracking systems, especially those developed for bees and ants such as BEEtag [28], bTools [18], or anTraX [29], preserve individual identity using unique barcodes or paint marking. Preserving identity has many benefits: specific individuals can be singled out before or after an experiment for targeted manipulation or analysis [19]. Coupling a marker-based system with pose estimation, as done in NAPS [30], represents a new category of ‘hybrid’ tracking systems that have so far been applied to the common eastern bumble bee *Bombus impatiens* [30,31].

**Figure 2:**
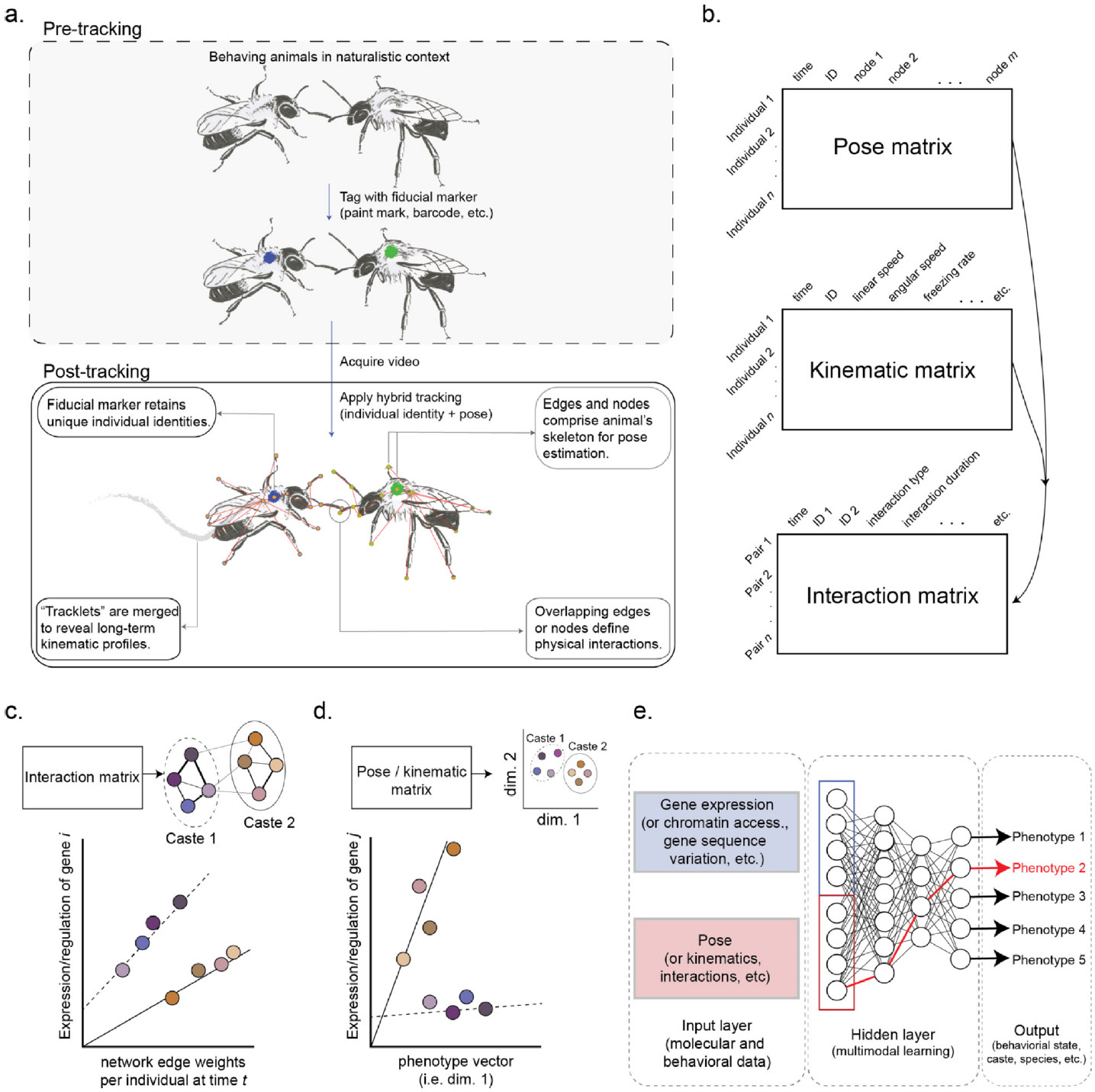
Integration of hybrid tracking and neuromolecular data sets. **(a)** Hybrid tracking brings together fiducial markers and pose estimation to allow for preservation of individual identity and flexible definition of an individual’s skeleton for the duration of an experiment, **(b)** thus generating high-dimensionality matrices containing everything an individual does. Pose data, which contain the position of each individual’s body over time, and kinematic data, which utilize the fiducial marker to quantify movement dynamics, can be coupled to define any type of physical interaction. Neuromolecular data can be integrated with automated tracking by comparing individual-level gene activity to continuous behavioral covariates such as **(c)** social interactivity, **(d)** phenotype vectors extracted from dimensionally reduced trait matrices, or **(e)** by deep learning with NNs. In **(c,** **d)**, ‘caste’ is assumed to be morphologically distinct or otherwise unambiguously characterizable, such that intercaste behavioral variation, as detected via automated tracking, refines molecular analyses.

Hybrid tracking allows for scalability without compromising spatiotemporal resolution: monitoring each individual’s pose affords users flexibility in exploring the mechanics of spontaneous and social behaviors (Figure 2a,b). The power of this concept should not be understated, as all observable behavior is composed of motifs organized in a specific order, as described by Lorenz and Tinbergen nearly a century ago [32,33]. Moreover, breaking down behavior into its fundamental units yields a ‘common currency’ that allows for powerful inter- and intra-individual comparisons, as well as across taxa [34]. Utilizing pose estimation from a hybrid tracking platform, supervised learning approaches such as SimBA [35], which learn to associate specific poses with user-defined behavioral patterns (i.e. aggressive postures, courtship displays, etc.) can automate the identification and quantification of specific behaviors over the course of an experiment. In contrast, unsupervised approaches do not require the predefinition of any specific behavior and instead learn stereotyped animal movement patterns, which a computer sees as specific configurations of pose over time. Tools such as Keypoint MoSeq [36] use unsupervised learning to identify the ‘syllables’ or stereotyped behavioral motifs that act as fundamental units of an animal’s behavioral repertoire.

Physical social interactions play a clear role in colony organization. For example, stereotyped antennal dueling in *Harpegnathos saltator* is predictive of reproductive success [37], and antennal ‘shivering’ patterns characterize subordinate behavior in *Odontomachus brunneus* [38]. These observations suggest that complex signals are communicated via the interaction dynamics of relatively simple anatomical structures, and we are still early in ‘decoding’ the mechanics of the physical interaction landscape. Early efforts utilized nestmate proximity in *Camponotus fellah* as a surrogate for interactivity [20], and newer approaches have capitalized on species-specific interaction modalities such as liquid food sharing (trophallaxis) interactions in honey bees [18]. By leveraging pose estimation, a user can define an interaction as the intersection of, for example, the nodes and edges that make up the antennae of two or more animals’ skeletons (Figure 2a, lower panel). The ability to flexibly design and quantify an interaction using automated methods is especially useful as social signals may be tissue specific: insect chemical profiles can vary by body part [39] and, as such, different types of interactions (i.e. head-to-head, head-to-thorax, etc.) may characterize different inter-active dynamics. A potential strategy for the future would be to perform unsupervised learning on instances of specific interactions (i.e. queen-worker, dominant-subordinate, etc.) to elucidate the relationship between movement dynamics and information exchange. Finally, we anticipate future interaction detection pipelines will also include automated detection of socially transferred materials, perhaps with a strategy for quantifying material transfer itself [40].

## Neuromolecular control of social behavior: steps toward integration

Both spontaneous and social behaviors have electro-physiological representation in the brain, and *in vivo* recordings are able to map specific behavioral outputs to individual neurons, neural circuits, or entire brain regions in insects [41]. Can the same be said of neuromolecular (i.e. genomic) activity? While profiling strategies such as RNA- and ATAC-sequencing are often used to assess gene expression and regulation, we are no longer restricted to whole-tissue or “bulk” approaches as advances in single-cell and spatial ‘omics now allow for resolution and throughput that profiles the brain one cell at a time [3], just as modern neurophysiology grants the researcher access to the firing profile of single neurons.

Sub-second kinematics and acute instances of stereotyped behaviors, especially in the context of behavioral syllables as described above, are unlikely to have strong molecular signatures that can be reliably detected on their own. This follows the logic of the ‘genomic action potential’ (gAP), a framework for understanding the ‘handoff of electrical to molecular activity in the excitable cells that compose the brain [42-44]. The gAP framework was initially proposed as a contrast to the electrical action potential (eAP), the mechanism by which neurons propagate information throughout the nervous system via millisecond-scale shifts in membrane potential [42]. The eAP, occurring after a neuron integrates synaptic input above a certain threshold, contributes to the generation of rapid behavioral responses but does not alone explain how animals use present information to adjust future behavior. The gAP framework, on the other hand, describes how both synaptic input and neuronal firing trigger subsequent gene expression events: while the singular eAP represents a digital ‘all-or-nothing’ output, the slower-acting gAP initiates molecular cascades that modify relevant cells or circuits to drive lasting changes in behavior [44].

Evidence of the gAP at play has classically been demonstrated via detection of immediate early gene (IEG) activity in the brains of vertebrates and invertebrates [45,46]. IEGs are usually transcription factors that transiently express in recently activated brain cells, serving to induce downstream transcriptional events such that new information is encoded to modify future neuronal activity. The timing, location, and magnitude of IEG activity in the brain may reflect the nature and context of a behavioral event and, as such, IEG localization and quantification is a powerful tool for mapping activity-dependent neural circuitry or cell types involved in a particular behavior. As in vertebrates, highly conserved IEGs, including *early growth response protein-1* (*egr1*) and *hormone receptor 38* (*hr38*), enable a careful dissection of behaviorally relevant neural circuitry with high spatial and temporal resolution [46]. Sustained activation of *egr1* and *hr38* occurs in the brains of actively foraging honey bees [47,48] and bumble bees (*Bombus ignitus*) [49] while more temporally and spatially restricted expression of these genes following a brief aggressive or caregiving interaction has revealed valence-encoding neurons in honey bees [50]. We anticipate IEG mapping will be greatly aided by advances in spatial transcriptomics, in which a given neuron’s molecular and anatomical identity will be quantifiable alongside its activation status across different behavioral axes.

Beyond IEGs and their downstream gene expression programs, prolonged behavioral states are composed of a suite of correlated traits (e.g. honey bee nurses, guards, and foragers; Figure 1). Unlike responses to brief social encounters [51,52], behavioral states tend to be associated with more persistent changes in gene expression and regulation [53-55] that together contribute to the production, signaling, and metabolism of neuropeptides and neurotransmitters that support variation in behavioral repertoire and responsiveness (i.e. the traits that make up a behavioral state). For example, transcriptomic profiling of novelty-seeking behavior in honey bee scouts uncovered variation in the expression of dopa-minergic, octopaminergic, glutamatergic, and GA-BAergic synthesis-related genes relative to nonscouts [56], and these results were also corroborated by a mass spectrometry–based analysis of a related search behavior [57]. While brain chemistry quantification has not yet reached the same throughput as ‘omics technologies, links between the two systems are similarly important to establish.

It is important to consider that, unlike electro-physiological data, molecular profiling of the brain at bulk or single-cell resolution requires destructive sampling: tissue must be removed and processed in steps leading up to library preparation, sequencing, and analysis. An advantage of most insects is that whole bodies can be flash frozen or directly placed in liquid nitrogen to ‘freeze’ molecular processes before being transferred to long-term cold storage. For many ants, bees, and wasps, the cuticle surrounding the brain can be chipped away in frozen conditions, revealing tissue that can be directly inputted to bulk or single-nuclei ‘omics methods. For single-nuclei sequencing technologies, which perform comparably to single cell–based methods [58], starting with frozen nervous tissue provides an advantage, as it evades the need for enzymatic disruption and mechanical dissociation of neurons, processes necessary for single-cell sequencing that can cause substantial stress and cell death. Furthermore, spatial transcriptomics methods currently allow for freshly dissected brains to be fresh frozen (i.e. frozen without a fixative) in a block of cutting media before long-term storage, thus preserving both expression profiles and tissue morphology. In the laboratory or field, these features are a tremendous boon for minimizing the time between observation, collection, and preservation for sequencing.

### Integrating behavioral and neurogenomic data sets.

At both bulk and single-cell resolution, RNA- and ATAC-sequencing experiments yield large count matrices of gene transcripts or accessible chromatin regions, respectively. In behavioral analyses, these matrices are typically used for comparing individuals that have been binned into discrete categories based on reproductive and/or morphological caste, task specialization, or behavioral display (i.e. nurse, forager, queen, etc.). Binning is a practical strategy but largely ignores interindividual variation, even in seemingly discrete contexts. For example, honey bee queens are morphologically and physiologically distinct from nonreproductive workers and express a different behavioral repertoire. Despite this extreme dimorphism, each queen’s behavioral and neuromolecular state is tied to her egg-laying status, which changes over the lifetime of a colony [59]. How can this be factored into a molecular analysis? And, more broadly, how should molecular correlates of behavior be identified with no clear ‘bin-able’ distinctions across individuals, as is the case in more simple eusocial societies with weaker reproductive division of labor?

One strategy is to model gene expression (or chromatin accessibility, genome sequence variation, etc.) on continuous behavioral covariates produced by automated tracking. This approach has been deployed via manual observations: Shpigler et al. [52] identified dozens of neural signaling-related genes, the expression levels of which correlated with the amount of time a honey bee nurse spent in contact with a larva [52]. Importantly, these contact-associated genes did not appear in a list of differentially expressed genes generated by comparing nurses to non-nursing controls. Variables such as frequency and duration of social interactions or number of interaction partners are readily generated by existing tracking platforms [19,22,53] and could be used to a similar effect (Figure 2c).

As large tracking data sets may contain many quantitative descriptions of individual- and group-level behaviors, similar dimensionality reduction techniques can be performed to extract behavioral correlates for regression analysis. For example, such an approach was used in honey bees to detect genetic variants associated with colony aggression: Avalos et al. performed multidimensional scaling on data from repeated quantitative assays on aggression and extracted a ‘phenotype vector’ that was then used to identify associated genetic variants [60]. Conceptually, phenotype vectors may be a valuable strategy for detecting molecular correlates of high-dimensional behavioral data (Figure 2d).

Machine learning, even in the earliest days of brain-wide microarray profiling in insects, has been a powerful, if underutilized, tool for predicting behavioral states such as nursing and foraging in honey bees [9], reproductive role in vespid wasps [61,62], and social polymorphisms in the sweat bee *Lasioglossum baleicum* [63]. These approaches could readily be extended to integrate the multifaceted data streams generated by the behavioral and molecular methods discussed above. Deep learning approaches such as NNs identify patterns or relationships between an input layer and an output layer. These analyses could simultaneously incorporate genotype, molecular phenotype (gene expression, chromatin accessibility, etc.), and latent representations of pose estimation data as input. The output layer, which represents the predictions or classifications made by the network, could then include caste, behavioral state, or even species (Figure 2e), tasking the NN with learning how each layer interacts to generate a given phenotype.

## Future directions

Moving forward, we anticipate automated tracking will allow multidimensional dissection of eusocial traits to facilitate closer examination of the different axes of behavior exhibited by the full range of social insects. Studies examining finer-scale variation in a range of traits linked to social complexity have revealed that lineages can evolve changes in these traits along different, independent trajectories [64]. Bumble bees are an ideal species to illustrate this point because colonies exhibit features of both simple and complex societies. For example, queens and workers are different in size, and the critical period for caste determination is established early in development, both hallmarks of more complex eusocial societies. However, there is limited evidence for allometric scaling across reproductive and nonreproductive castes, a trait often associated with more complex social forms, and although workers do not mate, a substantial proportion do contribute to colony reproductive output via the production of haploid eggs. Thus, the social traits of this species are distributed across different axes of social complexity. Automated tracking approaches that interrogate the multidimensionality of eusocial traits could provide a more powerful framework for mapping social phenotypes to molecular mechanisms both within this species and across the multiple origins of eusociality in Hyme-noptera [64,65].

Real-time automated analysis, in which data generated by behaving animals are immediately available to the investigator, is currently implemented in tracking tools such as SLEAP and DeepLabCut-Live! [66,67]. In proof-of-concept ‘closed-loop’ frameworks, specific behaviors trigger immediate neurophysiological feedback that rewards, punishes, or modifies how an animal acts in a given context. This could be an especially powerful means of uncovering the mechanisms that drive individual- and group-level behaviors in social insects, especially as *in vivo* neural monitoring and stimulation methods continue to develop for the tiny brains of freely interacting social insects [68,69]. Moreover, further development of tools such as NAPS [30] will allow for the real-time characterization of colony-wide social networks, meaning individuals occupying specific positions or engaging in certain behaviors in a network can be readily targeted for manipulation. Finally, we suggest a long-term goal for the social insect community: full characterization of the neural-to-genomic information flow for all insects in a colony, pairing real-time behavioral tracking with *in vivo* brain recording and genomic methods discussed here. Again, bumble bees are especially amenable to this goal owing to relatively large brain size, ease of barcoding and tracking, and flexibility of social environment.

We intend for this article to generate discussion regarding the definitions and classification of social insect behavior in light of advances in automated tracking and molecular profiling technologies. It is our hope that the ideas presented here, as well as decreasing costs of technology and computation, will facilitate the adoption of automated tracking among social insect researchers. The integration of these higher-resolution behavioral and neurogenomic tools should facilitate new levels of understanding about how genome dynamics modulate neurobiological function to shape the diversity and evolution of social behaviors.

## Data Availability

No data were used for the research described in the article.
